# Form and Function of Sleep Spindles across the Lifespan

**DOI:** 10.1155/2016/6936381

**Published:** 2016-04-14

**Authors:** Brittany C. Clawson, Jaclyn Durkin, Sara J. Aton

**Affiliations:** ^1^Department of Molecular, Cellular, and Developmental Biology, University of Michigan, Ann Arbor, MI 48109, USA; ^2^Neuroscience Graduate Program, University of Michigan, Ann Arbor, MI 48109, USA

## Abstract

Since the advent of EEG recordings, sleep spindles have been identified as hallmarks of non-REM sleep. Despite a broad general understanding of mechanisms of spindle generation gleaned from animal studies, the mechanisms underlying certain features of spindles in the human brain, such as “global” versus “local” spindles, are largely unknown. Neither the topography nor the morphology of sleep spindles remains constant throughout the lifespan. It is likely that changes in spindle phenomenology during development and aging are the result of dramatic changes in brain structure and function. Across various developmental windows, spindle activity is correlated with general cognitive aptitude, learning, and memory; however, these correlations vary in strength, and even direction, depending on age and metrics used. Understanding these differences across the lifespan should further clarify how these oscillations are generated and their function under a variety of circumstances. We discuss these issues, and their translational implications for human cognitive function. Because sleep spindles are similarly affected in disorders of neurodevelopment (such as schizophrenia) and during aging (such as neurodegenerative conditions), both types of disorders may benefit from therapies based on a better understanding of spindle function.

## 1. Introduction: Why “Spindle”? Generation and Termination of a Very Conspicuous Waveform

**Figure 1:**
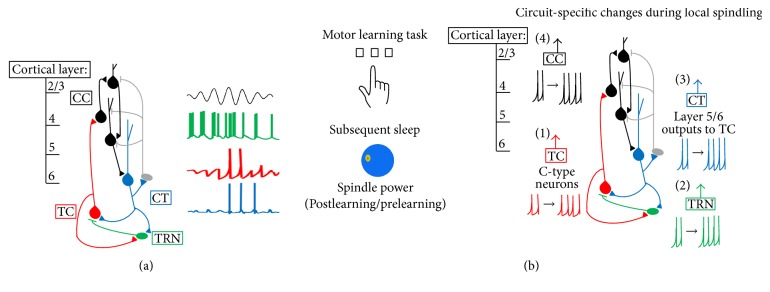
Spindles generation within the CT-TRN-TC circuit. (a) Sleep spindles are generated through reciprocal interactions of the TRN (*green*) and TC (*red*) neurons. Spindles can be initiated by excitatory input from CT (*blue*) neurons, which also synchronize spindle oscillations across the thalamocortical network. Representative neural firing patterns (*right*) show the order and organization of firing for CT, TRN, and TC neurons, respectively (adapted from [9]). As TC neurons fire, they provide input to both cortical and TRN neurons, and these populations phase-lock their firing. As cortical, TRN, and TC neurons desynchronize their firing, the spindle oscillation wanes. (b) The CT-TRN-TC circuit may also generate local spindles following waking experience. For example, training on a motor task leads to increased sleep spindles over the contralateral motor cortex during subsequent sleep (*left*; adapted from [10]). There are several possible mechanisms by which changes to CT-TRN-TC circuitry during waking could cause subsequent local spindle increases (*right*). TC projections may show changes to dynamics (e.g., firing rate or bursting) based on prior waking experience (1). Experience may alter TRN excitability or bursting during subsequent sleep (2). CT feedback may be altered by experience to increase synchronization or amplification of spindles (3). Finally, intracortical plasticity could amplify spindles locally (4).

*The Role of Thalamic Reticular Nucleus (TRN) Neurons*. Sleep spindles were the first features of sleep described in human EEG recordings [1, 2]. Their name reflects the waxing-and-waning nature of the 7–15 Hz spindle oscillation itself, which emerges, crests, and disappears over the course of approximately 1 s. These events occur at intervals during non-rapid eye movement (NREM) sleep. GABAergic neurons in the TRN play a critical role in the generation of these oscillations. Neurons in the TRN generate highly synchronized bursts at the outset of a spindle (Figure 1(a)). These bursts can be generated in NREM due to relative hyperpolarization in TRN neuronal membrane potential. This is due at least in part to reduced noradrenergic and serotonergic signaling [3]. Under these conditions, a relatively small amount of glutamatergic input (acting on ionotropic [primarily GluR4] receptors [4]) is sufficient to activate T-type calcium channels [5], the kinetics of which causes transient depolarization to spike threshold. After this calcium spike, the resting membrane potential undergoes afterhyperpolarization, mediated in part by calcium-activated, small-conductance potassium (SK2) channels (reviewed in [6]). Importantly, many TRN neurons are intrinsically capable of generating multiple cycles of membrane oscillations and firing bursts, which are facilitated by both Ca(V)3.3 T-type calcium channels [5] and Kv3-class voltage-gated potassium channels [7]. In the absence of ionotropic glutamatergic input, these oscillations can be initiated by mGluR activation and synchronized across the TRN via gap junction communication [8].

*The Role of Thalamocortical (TC) Neurons*. Despite the fact that some TRN neurons can generate spindle frequency oscillations in a seemingly cell-autonomous manner, sleep spindles are a network event. TRN bursts lead to transient GABA-mediated hyperpolarization in neighboring glutamatergic TC neurons elsewhere in the thalamus (Figure 1(a)). This hyperpolarization initiates a series of postsynaptic events in TC neurons: (1) firing becomes suppressed through GABA receptor-mediated inhibition, (2) due to membrane hyperpolarization, *I*_*h*_ (hyperpolarization-activated cation current) is activated and *I*_*t*_ (T-type calcium current) is disinhibited, and (3) due to activation of these currents, the membrane rapidly depolarizes, leading to a burst of action potentials [16]. This rebound burst results in a volley of excitatory input to TC targets in both the cortex and the TRN, where it sets off the next burst of firing in TRN neurons.

*The Roles of Corticothalamic Neurons*. While spindle oscillation generation relies on intrathalamic mechanisms, spindles can be driven from outside the thalamus. In particular, synchronous bursts of TRN firing are often initiated by excitatory input from layer 6 CT neurons, a major source of input to the TRN [17, 18] (Figure 1(a)). Indeed, some of the earliest descriptions of spindles noted their propensity to be initiated by sensory (e.g., auditory) input during sleep [1], consistent with initiation through either thalamic or cortical excitatory pathways.

Although the mechanism of pacemaking during spindles is well-understood at the cellular level, it is less clear how the conspicuous, spindle-shaped oscillation envelope that defines these events is generated. The waxing phase of spindle oscillations is generally thought to be caused by increasing activation of cortical neurons with each successive volley of excitatory TC input [19] (Figure 1(a)), leading to greater CT excitatory feedback, which synchronizes successive cycles of activity among neurons in the thalamus [6]. There are multiple theories regarding the waning and termination of spindles, each focused on changes in different parts of the CT-TRN-TC network. A number of studies have indicated that TC neurons gradually depolarize across successive spindle oscillation cycles. This eventually prevents activation of hyperpolarization-activated currents and associated burst firing [20, 21]. This effect may be due in part to disinhibition; the level of activity in TRN GABAergic neurons changes during the waning phase of spindle oscillations. TRN neurons become less active (firing fewer spikes per burst) with successive cycles of spindle oscillations [22]; this may be due in part to an increase in GABAergic signaling between TRN neurons as spindle oscillations progress [23, 24]. At the same time, TC neurons fire more spikes per burst across successive cycles. Afterdepolarization (mediated by Ca-activated *I*_*h*_ current) in TC neurons lasts several (i.e., 5–20) seconds, resulting in “refractory” periods between spindles and setting an upper limit on their frequency [21]. Indeed, Bal and McCormick [20] demonstrated that when *I*_*h*_ current is blocked in* ex vivo* slices, TC neurons generate spindle frequency oscillations which are continuous in nature, rather than spindle-shaped. Taken together, this suggests that excitatory TC input both increases and becomes less rhythmic as spindles wane and terminate. Feedback from excitatory CT neurons also appears to play a role in terminating spindles. Similar to TC neurons, CT neurons both increase their firing rate and fire in a less synchronous manner, across the waning phase [25]. This change in CT input could disrupt intrathalamic oscillations due to either desynchrony or neuronal depolarization (and thus disruption of thalamic bursting). Furthermore, increased CT input may play a role in termination by promoting greater intra-TRN inhibitory signaling (which would both inhibit TRN neuronal firing and desynchronize burst of firing between TRN neurons) with each successive cycle of the oscillation [23, 24]. These CT-TRN-TC changes seem to occur nearly simultaneously* in vivo*. Thus, while changes in each part of the circuit likely contribute to spindle waning and termination, it remains unclear which comes first in the sequence of events leading to termination.

## 2. Where Do They Come From, Where Do They Go? Spindle Topology, Synchronization, and Propagation

### 2.1. Global, Local, or Both?

Early reports suggested that spindles occurred independently in different cortical areas (i.e., frontal versus occipital) [2]. More recent studies on the topography of spindle occurrence (i.e., using high-density EEG) have indicated that, in humans and other mammalian species, spindles occur nearly simultaneously across many cortical areas [26–29]. However, these studies have demonstrated notable exceptions, in which “local” spindles occur independently at specific recording sites. Moreover, spindles occurring across the cortex are not completely coherent; the oscillations are not always phase-locked and do not always have the same intrinsic frequency. Rather, across multiple species [30–32], the frequency of oscillations shows an anteroposterior gradient, with spindles in frontal cortex showing a lower peak frequency (9–12 Hz) relative to those occurring at more posterior sites (13–15 Hz) [33] (although this does not appear to be true for mice, possibly due to differences in brain anatomy [29]). Furthermore, spindles do not initiate in perfect synchrony. Faster centroparietal spindles begin several hundred milliseconds earlier than slower spindles occurring in frontal cortex [27]. Experimental cortical stimulation is sufficient to generate “traveling” spindle oscillations that propagate outward from the stimulation site [28]. It seems likely that during natural sleep spindle oscillations, posterior-to-anterior propagation of spindles is mediated (at least in part) through CT connections, which support information transfer between neighboring cortical areas via the thalamus [34–36].

The more recent application of magnetoencephalography (MEG) to studying sleep oscillations has further complicated the picture of spindle generation and synchronization. Early studies [37, 38] demonstrated that spindle oscillations could be detected (either across the cortex or in a specific cortical location) in one modality but not the other (e.g., present in MEG and not simultaneously present in EEG). Subsequent data suggested that MEG frequently detects spindles that are not present in EEG, while the reverse is seldom true [39]. Additionally, within individual spindles, MEG waveforms are more variable between brain areas than EEG waveforms, with highly divergent oscillation phases, amplitudes, and periodicities among cortical recording sites [40].

### 2.2. Neural Underpinnings of Global and Local Spindles

Why are there such differences between MEG and EEG? MEG has better spatial resolution than EEG for superficial (cortical) brain activity, since magnetic fields suffer less distortion by the skull and scalp than electric fields. MEG and EEG may also have different sensitivities for detecting oscillations generated by anatomically different TC connections. Local spindle generation has been ascribed to highly focal projections of thalamic “core” (“C-type”) neurons, which send dense, spatially limited, and fast glutamatergic input to cortical layer 4. Such projections predominate in sensory cortical areas. In contrast, more diffuse “matrix” (“M-type”) projections provide slow glutamatergic input to large patches of layer 1 [41]. Available data suggests that MEG would be more sensitive to oscillations generated through the former pathway, while EEG would be more sensitive to oscillations generated through the latter [40, 42, 43].

While the general phenomenon of global versus local spindling has been primarily characterized as a cortical phenomenon, this may be due to methodological limitations. Most evidence of local spindling comes from high-density EEG and MEG recording, neither of which is useful for detecting thalamic signals. There is limited evidence that local spindles could be present the level of the thalamus, in TC or even TRN neurons. Decortication leads to gross desynchrony of spindles across TC sites* in vivo* [28]. Spindles recorded* ex vivo* in isolated thalamic slices are generally not synchronized [44], and stimulation of corticothalamic axons is sufficient to synchronize spindles within the slice [45]. Together, these experiments suggest that CT feedback is critical for synchrony. This is further supported by the recent finding that cortical EEG signals phase-lead thalamic EEG signals during NREM spindles* in vivo* [29]. However, because there are limited studies in which multisite thalamic recording has been carried out* in vivo*, it remains unclear how coherent thalamic spindle oscillations are during natural sleep or whether “local” oscillations are present.

Given all of the contributions of corticothalamic, intrathalamic, and thalamocortical circuitry to maintaining coherent sleep spindles, what circumstances give rise to local spindles? The anatomy of TC and CT projections plays a role: spindles are coherent between TC nuclei and the cortical areas they project to, but not between thalamic and cortical sites which are not functionally connected [46]. However, it remains unclear why they should occur synchronously in two cortical areas at certain times, and asynchronously at others. What is clear is that spindles can be more pronounced in specific cortical areas based on prior (wakeful) activity in those areas. This can be seen, for example, following training on a visual texture discrimination task, in which psychophysical discrimination thresholds decrease for a specific retinotopic area of the visual cortex. This retinotopically specific improvement is dependent on posttraining sleep [47, 48], and during posttraining sleep MEG spindle power is significantly increased in these trained retinotopic areas [49]. To date, evidence for “use-dependent” local spindle generation has primarily come from recordings in primary sensory or motor cortical areas, where C-type TC projections are most abundant.

There are several physiological reasons for why circuit activation in sensory and motor cortices might preferentially lead to local spindling, which are by no means mutually exclusive. One possibility is that C-type TC relay neurons projecting to these cortical areas show some hysteresis of activity or excitability, which translates to modality-specific changes in their subsequent dynamics in the hours following learning (Figure 1(b),* red arrow*). Indeed, experience-dependent changes in both membrane excitability and auditory responses have been recorded in TC neurons following behavioral conditioning to specific sounds [50]. A second possibility is that TRN neurons, which show topography similar to TC and cortical neurons [51, 52], show changes in firing properties, bursting, or intrinsic excitability based on experience-dependent alterations in input from thalamic and cortical neurons (Figure 1(b),* green arrow*) [53]. A third possibility is that the CT input returning* from* sensory and motor areas (i.e., those receiving C-type TC input [54]) is altered by learning in a manner which promotes local spindle amplification, or greater local spindle coherence (Figure 1(b),* blue arrow*) [45]. Plasticity of CT synapses on sensory relay neurons is well described [55, 56]. Importantly, CT feedback to thalamus could also vary as a function of cortical location, in a manner analogous to (and indeed paralleling) C- and M-type TC pathways. As discussed above, CT feedback from layer 6 sends input to both TC and TRN neurons. A subset of layer 5 neurons also send input to thalamus, but in contrast to layer 6 neurons, there is no clear evidence that these neurons send collaterals to the TRN [57, 58]. Depending on the cortical region, layers 5 and 6 CT projections from the same cortical area can partially overlap within the thalamus (potentially even sending input to the same TC neurons) [59], send input to distinct subregions of the same thalamic nuclei [60], or send input to completely different thalamic nuclei [57]. A fourth and final possibility is that intracortical plastic changes, initiated by sensory or motor experience, lead to subsequent changes in spindling via increased postsynaptic responses to TC input [61, 62] or increased CT feedback to the thalamus (Figure 1(b),* black arrow*) [63, 64].

## 3. So… Why Spindle? The Function of Spindles in Thalamocortical Circuits

### 3.1. The Relationship between Learning and Spindles

The phenomenon of local spindling has opened a window for better understanding of relationships between spindle oscillations and brain function. There is abundant evidence that local increases in spindling following specific types of learning (e.g., sensorimotor) may have important implications for subsequent cognitive function and performance. For example, training human subjects on a left-hand motor task leads to a subsequent, sleep-dependent improvement in task performance. This improvement is proportional to both EEG spindle density and lateralization of spindle power recorded at sites overlying right (contralateral) motor cortex (Figure 1(b)) [10]. Similarly, training on verbal and visuospatial memory tasks leads to overnight improvements that correlate with post-training NREM spindle numbers at left frontal and parietal EEG recording sites (areas associated with learning on these two tasks), respectively [65, 66]. Analogous lateralized changes in spindle density and amplitude are seen in sleep during odor-based cueing of visual information presented to specific visual hemifields during prior waking experience: odors paired with images presented to the left hemifield in waking evoke spindles in right visual cortex when presented again during subsequent sleep, and vice versa [67]. Recently, it was shown using a combination of high-density EEG and fMRI during postlearning sleep that local spindles lead to selective reactivation of brain areas previously engaged by the learning task [68, 69]. This reactivation included temporally coordinated increases in activity between the hippocampus and specific cortical areas engaged by learning a particular task [68]. Cumulatively, it seems clear that local spindles may either directly promote adaptive, circuit-specific plasticity following learning or be a highly reliable biomarker of such processes [62].

### 3.2. Do Spindles Drive Synaptic Plasticity?

Understanding the function of spindle oscillations in brain plasticity has major implications for human performance. Many years after Loomis noted that spindle occurrence was highly variable between subjects [1, 2], various spindle features have been found to correlate with general IQ measures in both children and adults [70–72]. As is true of any correlation, it remains unclear whether this indicates that spindle oscillations directly promote overall cognitive function, or rather whether optimal thalamocortical circuit function (and thus increased spindling) results from other processes that affect cognitive function. However, the numerous studies linking spindles to sleep-dependent improvement on new tasks suggest that the former relationship is plausible. A limited number of studies have found evidence that patterns of thalamocortical activity associated with spindles could lead to synaptic plasticity. One seminal study by Rosanova and Ulrich [73] used a pattern of neuronal activity recorded from rat somatosensory cortex during spindle oscillations* in vivo* to drive presynaptic activity in connected pairs of cortical pyramidal neurons (in layers 2/3 and 5, resp.). They found that this naturalistic activity pattern, repeated at intervals that mimicked the frequency at which spindles occur during NREM, could reliably evoke postsynaptic long-term potentiation (LTP). They also found that artificially generated bursts, generated presynaptically at spindle frequency (i.e., 10 Hz), evoked a similar form of LTP. Similarly, repetitive transcranial magnetic stimulation (rTMS) applied to the cat primary visual cortex (V1) at 10 Hz (but at not lower frequencies) over a period of minutes could elicit potentiation of visually evoked potentials [74]. Thus, available data indicate that simple patterns of activity occurring at spindle frequency are sufficient to induce potentiation in cortical synapses* in vivo.*

Studies using* in vivo* paradigms in which synaptic plasticity is induced by novel waking experience (e.g., novel sensory or motor experience) indicate a potential role for sleep spindles in promoting adaptive synaptic changes. Two recent examples come from the visual systems of cats and mice, in which novel visual experience leads to subsequent, sleep-dependent changes in thalamocortical circuit function. Both forms of plasticity are mediated by potentiation of glutamatergic synapses in the primary visual cortex (V1), leading to enhanced responsiveness to visual stimuli previously experienced in wakefulness. The first example, ocular dominance plasticity (ODP) in juvenile cat V1, is initiated by a very brief period (on the order of a few hours) of monocular visual experience. ODP takes the form of enhanced responsiveness to spared-eye visual stimulation in V1 neurons and is significantly enhanced by sleep in the hours following this experience [75]. Sleep-dependent ODP enhancements were recently shown to be mediated by LTP-associated mechanisms in V1 [76] and were proportional to phase-locking of V1 neurons' firing with NREM spindle oscillations in the hours following experience [77]. The second form of plasticity, orientation-specific response potentiation (OSRP) in adult mouse V1, is initiated by brief exposure to a patterned visual stimulus such as an oriented grating [78]. It is expressed several hours after this experience, as a selectively enhanced visually evoked response in V1 to stimuli of the same orientation. Available data suggests that OSRP is mediated by LTP-like changes at TC synapses in layer 4 [79], and data from our lab have shown that these changes occur during sleep in the hours following visual experience [80, 81]. Intriguingly, like sleep-dependent ODP, response changes during OSRP are also proportional to phase-locking of V1 neurons' firing with spindle oscillations in the hours following experience [80]. Taken together, these studies show that from the early development of sensory responses through adulthood, NREM spindles can support strengthening of cortical synapses in an adaptive manner, promoting enhanced neuronal responses to behaviorally relevant sensory stimuli.

The cellular mechanisms by which spindle frequency oscillations (versus network oscillations at other frequencies) promote synaptic strengthening* in vivo* are still a matter of speculation [82, 83]. However, the pattern of activity used to mimic spindle-associated activity* in vivo* (i.e., bursts of presynaptic activity occurring at approximately 10 Hz) [73] is similar to theta-burst stimulation (TBS) protocols used to induce LTP at glutamatergic synapses [84]. A great deal is known about cellular mechanisms mediating TBS-induced LTP in the neocortex [85–87] and the hippocampus [88]. Intriguingly, 10 Hz stimulation in the form of single spikes (instead of bursts) has the opposite effect on glutamatergic synapses, inducing long-term depression (LTD) [89]. Thus, one possibility is that, by generating 10 Hz inputs in a large population of thalamic inputs to the cortex, weak inputs (those generating only ≤1 spike/cycle) further weaken their connections with postsynaptic cortical targets, while stronger inputs (those generating bursts of spikes) strengthen connections with their postsynaptic partners. Thus, it is possible that, in certain conditions, spindles may also promote the preferential downscaling of weaker synapses during sleep [90].

Based on the available data, there appears to be a strong link between NREM spindles, plasticity in thalamocortical circuits, and cognition. How can this knowledge inform our understanding of early nervous system development, plasticity through childhood and adolescence, and cognitive dysfunction and neurodegeneration in the aging brain? The remainder of our discussion will focus on these issues, emphasizing spindle phenomenology and potential function across the lifespan.

## 4. Sleep Spindle “Precursors” in the Developing Brain

Studies of sleep across the lifespan of humans and rodents [91, 92] have demonstrated that sleep architecture changes dramatically across early postnatal development. In particular, both total sleep time and the relative proportion of REM to NREM sleep decrease dramatically between birth and adulthood. Because of the limitations of early EEG and behavioral techniques on which early studies in the field were based, until recently very little was known about pre- and perinatal sleep architecture and brain activity. Thus, almost nothing was known about how the activity patterns we associate with REM and NREM initially develop. However, recent studies using surface EEG in preterm and full-term neonates have allowed new insights into how sleep features emerge at early stages of brain development.

**Figure 2:**
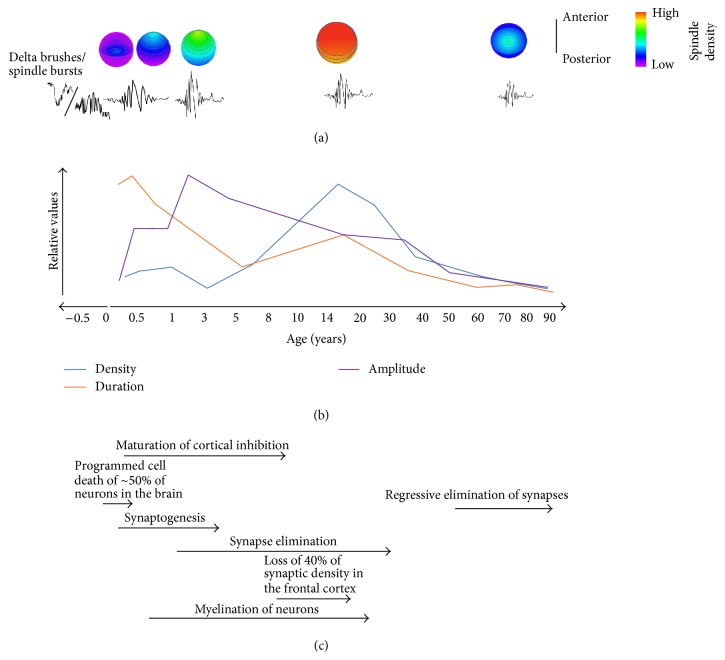
Changes in spindle form across the lifespan. (a) Heat maps depict topographical spindle density during early development, adolescence, and aging. Spindles are initially seen over central areas of the brain and gradually develop over frontotemporal areas during the first year of life [11]. During adolescence, density reaches a relative maximum with equal distribution across frontal, central, and parietal leads. In aging, there is a return to the same pattern seen earlier in development, with highest density at central leads [12]. Below the heat maps are representations of spindle morphology at these ages. (b) Sleep spindle density (*blue trace*) increases throughout early development, peaking during puberty and steadily declining from adolescence to old age [13, 14]. Duration of sleep spindles (*orange trace*), on the other hand, peaks early in life, and then generally declines over the lifespan. Spindle amplitude (*purple trace*) is relatively small early in development, increasing to maximum values over the first year of life, and then steadily declines until old age [15]. (c) Neurodevelopmental milestones.

EEG features termed “delta brushes” are the dominant pattern of activity seen in premature infants and* in utero* (Figure 2). They consist of spindle-like oscillations of 8–20 Hz, riding on top of delta (0.3–1.5 Hz) waves. Prior to 28 gestational weeks, they are found exclusively at central areas, but from 28 weeks to term, they can be seen at central, occipital, and parietal areas [93]. These oscillations can occur during any stage (sleep or waking); however, as the brain matures they become restricted to sleep [94]. Topographically, they tend to occur independently over the two hemispheres, possibly due to the absence of interhemispheric connections early in development. Delta brushes diminish in voltage and density by 9 months gestation and thus are predominantly studied in premature human infants [94]. Little is known about how delta brushes are generated, although they can be triggered by sensory input in a manner similar to spindles in the adult brain [95]. This suggests that delta brushes can be initiated by sensory-evoked activity in the same circuits that generate spindles in the fully developed brain.

To understand the mechanisms generating delta brushes and their potential function, many groups have examined an analogous oscillation in animal models, the so-called “spindle bursts.” Spindle bursts occur during developmental periods comparable to those in humans in which delta brushes are detected [96, 97] and comprise a similar oscillation frequency band (5–25 Hz). Spindle bursts, like delta brushes, occur locally and primarily during sleep [98]. They may also be initiated in a manner similar to both delta brushes and adult sleep spindles. Neonatal rats exhibit myoclonic twitches, after which spindle bursts occur in contralateral somatosensory cortex [98]. Spinal cord transection significantly decreases but does not eliminate spindle burst activity. Thus, like delta brushes, spindle bursts can be elicited by sensory stimulation, although external input is not required for their initiation. The tight temporal relationship between spindle bursts and myoclonic twitches and the fact that spindle bursts can be elicited by cutaneous stimulation have led researchers to speculate that they may provide sensory feedback to the developing somatosensory and motor cortices [97–99]. Such feedback may serve an important function for proper topographic mapping of these cortices during development. While the precise cellular mechanisms mediating this process are still unknown, one possibility is that (through LTP-like mechanisms) spindle bursts lead to functional activation of silent thalamocortical synapses. In fact, from postnatal days 2–5 in rats, a significant portion of thalamocortical synapses are silent. Isaac and colleagues [100] demonstrated that LTP could first be elicited at silent thalamocortical synapses in brain slices at postnatal days 3–7 (a period in which spindle bursts are frequent* in vivo*) but not at postnatal day 8 (when spindle bursts are rarely seen). Spindle bursts could also facilitate somatotopic mapping in other ways, for example, through plasticity of inputs to thalamic relays, or through plasticity of intracortical circuitry. It is tempting to speculate that, like spindle bursts, delta brushes may promote adaptive plasticity in synaptic connections. Delta brushes appear around the time that sensory-driven thalamocortical synapse formation is taking place, approximately 31–34 weeks of gestation [101]. At this developmental stage in humans* in utero*, generalized body movements begin to occur [102]. It is possible that delta brushes serve a similar purpose to topographically wire somatosensory cortex using sensory feedback following body movement. Consistent with this idea, Milh et al. [95] demonstrated that hand and foot movements in premature infants (31–33 weeks gestational age) preceded delta brush occurrence in developing somatosensory cortex; these brushes are local in nature, and occur predominantly contralateral to the movement. Hand and foot stimulation likewise elicited delta brushes in these cortical areas. Taken together, these findings support a role for delta brushes in the human brain* in utero* that is analogous to spindle bursts in animal models in early postnatal development; that is, in somatotopic cortical mapping [95, 97]. Recent studies in both animals and humans have sought to understand the role of spindle bursts and delta brushes in the development of other sensory cortical areas. Spindle bursts occur either spontaneously or are evoked by stimulation in visual cortex in rats [103, 104] and ferrets [105, 106], as well as barrel cortex in rats [107]. Likewise, delta brushes may play a role in human visual cortex development [108] and are seen in temporal areas following auditory stimulation [109].

How similar are these early oscillations to spindles in the mature brain? Spindle bursts in animal models and delta brushes and sleep spindles in humans are similar frequencies, although delta brushes are more likely to associate with delta frequency oscillations [94]. Furthermore, the topographies of delta brushes and sleep spindles throughout development are similar, each starting with a central distribution and spreading to other cortical areas as the brain matures [11, 93]. Delta brushes are also believed to be thalamocortical in origin, similar to sleep spindles [109]. Recent work by Yang et al. indicates that a fraction of spindle bursts in barrel cortex are dependent on the ventral posterior medial (VPM) nucleus of the thalamus [110]. Finally, spindle bursts, delta brushes, and sleep spindles may all play a functional role in synaptic plasticity [9, 73, 77, 80, 97–100]. Despite these similarities, it is unclear if spindle bursts or delta brushes can be called sleep spindle precursors in animal models and humans, respectively. After all, there is an unexplained two month gap between the disappearance of delta brushes and the appearance of the first sleep spindles in humans [94] (see below). More work exploring the mechanistic underpinnings of these early oscillations is necessary before it can be said whether or not these oscillations are truly sleep spindle precursors. Despite these reservations, delta brushes in humans and spindle bursts in animal models may represent the earliest instances of “spindle-like” oscillations playing a role in, or serving as a reliable biomarker for plastic changes in the brain.

## 5. Developmental Onset and Function of Mature Spindles

### 5.1. Phenomenology of Spindles across Childhood

In contrast to delta brushes and spindle bursts, mature sleep spindles do not occur for several weeks following birth [94]. Available data suggest that between 6 weeks and 3 months of age, mature spindles appear and gradually increase in number and duration (Figure 2) [13, 111, 112]. The earliest spindles appear primarily around central leads (i.e., over the central sulcus dividing primary motor and primary somatosensory cortices) of EEG recordings and are bilaterally synchronous [11]. By 4 months of age, there is greater distribution to frontotemporal regions, with higher levels of asynchrony in those areas; by 12 months, these spindles become more synchronous, possibly due to the increase in cerebral cortex white and gray matter by 88% during the first year of life [11, 113]. In fact, synaptogenesis, apoptosis, and myelination, which are all crucial in the formation of functional neural circuits, continue from infancy through adolescence [114].

Childhood and adolescence are also periods of substantial change in spindle frequency, distribution, and power. Over the course of development, the occurrence of both slow and fast spindles increases [115]. During childhood and adolescent maturation of frontal gray and white matter, there are corresponding changes in spindle power in the slow (9–12 Hz) frequency band which predominates in adults in frontal regions [116]. Frontal (slow) spindles become more prominent with age, with a sudden increase in frequency during puberty, despite an overall decrease in power [117–119]. In contrast, there is very little age-dependent change in the power of centroparietal (fast) spindles across this same developmental period [118]. Topographically, spindle amplitude becomes greater in anterior regions, and decreases in posterior regions in children 7–11 years of age [120] (Figure 2). In children and adolescents, spindle density, and frequency also vary overnight as a function of time [120, 121], with spindles becoming faster over successive NREM cycles [120].

### 5.2. Spindles and Cognitive Ability

Though these changes in spindle activity and topography have been well documented, until recently, there was a relative paucity of literature describing the role of spindles in postnatal cognitive development (i.e., in children and adolescents). While studies in adults have found positive relationships between NREM spindle occurrence and IQ [72, 122], findings have been less consistent in children. A recent study found a positive correlation between total spindle power in NREM and the nonverbal IQ in children aged 9–12 [71]; however, this relationship seems to vary widely depending on the frequency band under study. “Fast” and “slow” sleep spindles correlate negatively and positively, respectively, with IQ measures. Faster spindle oscillations are negatively correlated with IQ [71], working memory, fine motor skills, planning ability [123], and perceptual reasoning [71] in children. In contrast, slow spindles show the opposite relationship to cognitive abilities. Doucette and colleagues examined younger (i.e., preschool) children and found a positive relationship between simple reaction time task performance and slow spindle power [115]. Importantly, many of the above studies have focused on “fluid intelligence” rather than “crystallized intelligence” (e.g., skill learning and episodic memory consolidation). The former appears to rely more on genetics than the latter, which seems to benefit substantially from both experience and sleep [124]. Based on this distinction, Gruber and colleagues have suggested that a common heritable factor may affect both the speed of spindle oscillations and measures of fluid intelligence [120].

More recently, experiments have begun to address the role of sleep spindles in building crystallized intelligence during development. Unlike adults, children under 5 sleep in biphasic or polyphasic patterns, taking naps in addition to overnight sleep. Spindles are hypothesized to drive improvements in learning seen after a short nap in preschoolers [125]. These midday naps may be particularly important for preschool-aged children, since parietal and temporal cortices mature around this time and prefrontal cortex is beginning to undergo plasticity based on experience [126]. Kurdziel and colleagues found that sleep spindle density during a nap was positively correlated with sleep-dependent (i.e., postnap, prenap) improvements in children's performance on a visuospatial task [125].

A good understanding of the differences between spindles' role in children versus adults is still lacking. What is clear is that spindles in children are particularly associated with intrinsic measures of intelligence, and often these correlations are negative. In adults, these correlations are frequency positive. One possibility to explain these differences is the need for refinement and pruning of synapses in children. Myelination, synaptogenesis, and apoptosis occur throughout childhood and adolescence. Chatburn and colleagues have speculated that negative correlations represent the immaturity of the thalamocortical network and intracortical connections [123]. Though studies examining spindle function in children are lacking, there are even fewer studies in adolescents. This represents a significant gap in the literature, as puberty and adolescence are times of significant change in both body and brain development, marking the transition from childhood to adulthood.

## 6. Sleep Spindles and Disorders of Neurodevelopment

### 6.1. Down Syndrome, Cortical Malformations, and Autism Spectrum Disorders (ASD)

The relationship between spindles and brain development has been examined in patient populations where cognitive development is delayed or disrupted [127, 128]. For example, in children with Down syndrome, there is a significant delay in the developmental onset of identifiable spindle activity, spindle density is significantly reduced, and development of interhemispheric synchrony of spindles is delayed [127]. These differences are likely due to the numerous neurodevelopmental abnormalities associated with Down syndrome. For example, children with Down syndrome show decreased proliferation of neuronal progenitor cells during gestation, decreased mature neuronal numbers overall, decreased dendritic spine density, changes in LTP and LTD, and early-onset neurodegeneration when compared with typically developing children [129–131]. Patients with cortical malformations, in which gray matter and (to a lesser extent) white matter do not develop properly, have decreases in bilaterally occurring spindles relative to controls [132]. Spindle occurrence in these patients is less frequent in the hemisphere exhibiting the malformation, indicating disruption of thalamocortical pathways to generate spindles. Children with ASD also show significantly reduced spindle density during sleep [133, 134]. Thus, it seems that spindle density could serve as a generalizable biomarker for disorders of neurodevelopment.

### 6.2. Childhood Depression

Some disorders emerge only later in development, as children's brains mature; available data indicate that these disorders may also have features related to deficits in sleep behavior generally and sleep spindles in particular. For example, a substantial number of studies have described decreases in overall sleep time and disrupted sleep architecture in early-onset major depressive disorder (EO-MDD), which is highly prevalent in both children and adolescents [135]. Recently, Lopez et al. [121] demonstrated that both children and adolescents diagnosed with EO-MDD and those undiagnosed but at high risk for EO-MDD (based on a significant family history of MDD) showed reduced spindle density across the night compared to those with neither a diagnosis nor a family history. Because spindle reductions have been less consistently found in adult MDD patients [136, 137], one possibility is that a reduction in spindles at this earlier developmental stage (i.e., when the brain is more plastic) has a more pronounced effect on cognitive function (resulting in earlier symptom presentation).

### 6.3. Schizophrenia

Researchers have also known for some time that sleep disturbances are common in schizophrenia patients. Spindles are reduced in density, amplitude, and duration in schizophrenia patients who are either medicated [138–140] or drug-naïve [141]. It is still unclear how these changes relate to cognitive and behavioral dysfunction in schizophrenia. Since patients typically first present with symptoms of schizophrenia during adolescence [142], decreased spindling during adolescent development may be a diagnostic biomarker for these patients [141]. It is possible that decreased spindle density in schizophrenia patients is due to either structural or functional abnormalities in TC signaling, for example, due to changes in the number of TC neurons [143] or in the balance between intrathalamic excitatory and inhibitory signaling [144]. There is evidence of both GABAergic signaling deficits [145] and abnormal glutamatergic signaling in the thalamus of schizophrenia patients [146, 147]. Interestingly, decreases in thalamic volume in schizophrenia correlate with both decreases in NREM spindles and cognitive dysfunction [143]. One intriguing possibility is that treatments aimed at increasing sleep spindles in schizophrenic patients could have clinical value [141]. Recently, hypnotics which increase spindle numbers (such as eszopiclone) have been tested as a treatment for cognitive dysfunction in schizophrenia [141, 148]. One study assessed overnight improvement on a motor sequence task in patients trained in the evening and subsequently treated with either eszopiclone or a placebo. Eszopiclone significantly increased NREM spindle counts, and while there was no significant effect of treatment on overnight improvement on motor performance, spindle counts in both the placebo and eszopiclone groups correlated with overnight improvement on the task [148]. This strategy holds promise, particularly as a longer-term (i.e., nightly versus one-time) adjuvant treatment for cognitive dysfunction in schizophrenia.

## 7. Aging Alters Architecture: Sleep Spindle Changes in Normal Aging

Aging adults self-report sleep issues at a greater frequency than any other age group [149]. While the general population has an insomnia rate of up to 48%, the aging population reports a prevalence of up to 65% (reviewed in [150]). In aging, sleep is altered in multiple capacities including timing, duration, architecture, and quality. The elderly population experiences a circadian phase shift in which they wake up earlier and go to sleep earlier than younger adults, with decreased duration of sleep and increased napping [151]. The sleep aged adults do get is more fragmented, with increased arousals and more frequent transitions between sleep states [152]. Total time spent in both REM and deep slow wave sleep (SWS) decreases, while lighter NREM sleep increases in duration [153]. The density of both K complexes and sleep spindles decreases [15]. NREM spectral power decreases in alpha (relaxed wake), delta (deep sleep), theta (consciousness), and sigma (spindle) bands. Sleep macroarchitecture across the night also changes dramatically. In young adults, across the night, SWS decreases in duration and spindle occurrence decreases as the night goes on. This is thought to reflect homeostatic changes in NREM sleep after wakefulness, leading to greater SWS and spindle occurrence at sleep onset and decreasing SWS and spindle amounts across the night. Aging alters this process, making SWS duration and spindle occurrence more homogeneous across the night [154, 155].

Age-dependent changes in spindle density, amplitude, duration, and topography have been extensively characterized using EEG (Figure 2) [15, 156, 157]. All of the reported sleep spindle changes appear to be progressive with increasing age [14, 158]. For example, spindle density progressively decreases (relative to density in young adults) across middle age and into old age [14]. Three features of age-dependent spindle decline are noteworthy, where they may illustrate the neurobiological changes underlying this process. First, these changes are not homogenous across the cortex; rather, decreases in spindle density at older ages are most pronounced at frontal and occipital sites [12]. This progressive topographical change is actually the reverse of what appears to happen during development, in which density in these areas gradually increases relative to the rest of the cortex (Figure 2). This may relate to progressive neurodegenerative changes that preferentially affect these cortical areas with aging [159]. It also appears that slow and fast spindles are differentially affected with aging. There is general agreement that NREM spectral power in the fast spindle range (i.e., within 13–15 Hz) gradually, but dramatically, decreases with age [155, 160]. However, it is unclear whether slower-frequency (9–12 Hz) spindles are similarly affected. Finally, it is possible that global and local spindles are differentially affected with advanced age. A loss of local spindles may indicate either a selective age related change in the CT-TRN-TC circuitry mediating local spindles or general decline in neuroplasticity with aging. Recently, a study by Huupponen and colleagues [161] found a (nonsignificant) trend of increasing prevalence of global versus local spindles with increasing age. While the spatial resolution was limited in this study (only six EEG channels were used) future studies using higher density EEG and MEG may clarify how aging affects the topology of spindle occurrence, and local spindles induced by prior learning are affected with aging.

Not everyone is equally affected by spindle changes with age. For example, in younger and middle aged subjects, there is a clear gender difference in spindling, with women having higher spindle density; in aging this difference decreases [15]. Across-the-night changes in spindle density are also altered in older subjects [155–157]. Even when differences in age and gender are accounted for, there is still high variability, with some adults being much less affected by age than others [162].

## 8. What Are the Effects of Spindle Changes as We Age?

There have been two main focuses for understanding the functional effects of age-related changes in sleep spindles: (1) possible changes in sleep architecture and (2) potential changes in cognition. In fact, the earliest study looking at the effects of spindle changes examined both. Guazzelli and colleagues found no correlation between spindle density and either the number or the duration of bouts of wake after sleep onset in older subjects [156]. Similarly, Nicolas et al. found no interaction between sleep spindle characteristics and overall sleep efficiency in aging subjects [14]. Guazzelli et al. also examined the potential relationship between spindle decrease and cognitive decline. They found no correlation between overall verbal or performance IQ and spindle changes with aging. However, studies using more specific cognitive measurements (e.g., with tests of motor learning and episodic memory) found consistent correlations between preservation of spindles with increasing age and preservation of cognitive functioning [163, 164].

Many age-related pathologies present with cognitive decline, including Alzheimer's and Parkinson's diseases. In these two syndromes, further decreases in spindle density (beyond that seen in normal aging) have been observed [165, 166]. The most dramatic reductions—and even a complete loss of sleep spindles—have been reported in the most severe cases of dementia [167, 168]. Alzheimer's disease patients who presented with greater mean intensity and number of fast spindles performed better in an episodic memory (story recall task) than patients who showed lower spindle numbers and intensity [165]. Similarly, a decrease in spindle density and amplitude has been observed in Parkinson's disease patients who experience dementia relative to those who do not. These decreases occurred to a greater extent in posterior areas, with prominent decreases at central, parietal, and occipital derivations. The strength of the decrease in posterior areas was correlated with a decrease in visuospatial capabilities [166]. Interestingly, a similar spindle-cognition relationship has been observed in a premature aging syndrome (Mulvihill-Smith syndrome), with a complete loss of NREM spindles associated with cognitive deficits [169].

## 9. Why Do We Lose Spindles as We Age?

There is a large gap in our understanding of the neurophysiological changes that cause declines in spindling with age. However, some speculations can be made. Changes in brain anatomy with aging are often referred to under the umbrella of the “shrinking brain phenomenon”: as senescence proceeds, cortical volume decreases [170]. This occurs in an anterior to posterior gradient, with both gray and white matter [171]. Decreases in gray matter are concentrated in prefrontal cortex, alongside the inferior frontal and insular regions, and the inferior parietal cortex [170, 172, 173]. White matter is most reduced with aging in medial frontal and medial lateral regions [174, 175]. These alterations may offer explanatory value for region-specific decreases in spindling with aging. In line with this, decrease in spindles in aging has been correlated with increased sulcal atrophy [156]. Additional age-related thalamocortical circuit changes may explain overall decreases in spindles. For example, the thalamus shows a significant decrease in volume in aging, and its connectivity with the cortex is altered (reviewed in [176]). This change parallels structural changes associated with reduced spindling in the brains of schizophrenia patients described above. Furthermore, T-type calcium channel (Cav3.1) expression decreases as a function of age [177]. This latter change could translate into a fundamental deficit in the generation of spindle oscillations, independent of anatomical changes to TC circuitry. Finally, given that melatonin signaling enhances activity in the 11–14 Hz frequency band [178–180] and melatonin levels decrease significantly with age [179], this may account for both the reduced occurrence of spindles and the upward shift in spindle frequency in aging.

## 10. Summary and Future Directions

Previous work clearly defined that spindles evolve across the lifespan and that changes in spindle activity are correlated with changes in cognition. However, a major gap in our knowledge remains with regard to causality. We currently do not know for certain whether (and how) network activity in wakefulness promotes local spindle activity. Neither do we know whether (and how) global and local spindles contribute to thalamocortical network plasticity and cognitive functions associated with this plasticity. The use of new tools, such as pharmacogenetics, optogenetics, or transcranial magnetic stimulation, to alter spindle generation or density should clarify our understanding of the relationship between spindle form and function.

A majority of the literature examining sleep spindle function in humans has focused on adult populations. However, broadening our understanding of these oscillations throughout the lifespan may illuminate spindle functions that are not apparent in healthy adults. Infancy, childhood, and adolescence are times of dramatic change in the brain, characterized by widespread synaptogenesis, myelination, and synaptic pruning. During these years, spindle amplitude, duration, density, frequency, and topology change. Understanding the relationship between spindle form and function during these transitions has implications for a variety of neurodevelopmental disorders in which spindle activity is altered.

With advancing age come dramatic alterations in brain architecture, cognition, and sleep patterns. Many of these are the reverse of changes seen during development. Both healthy age-related and pathological changes in the brain cause decreases in brain volume and synaptic connections. Decreases in sleep and sleep spindles during this time are likely caused by structural alterations in thalamocortical circuitry. However, augmentation of spindles may be useful for mitigating deficits seen with cognitive aging.

In conclusion, sleep spindles change over the course of a lifetime, in parallel with the multitude of changes in the developing and aging brain. Spindles may serve different functions throughout the lifespan. Future studies focused on age-specific brain anatomical and functional differences will ultimately help us understand these functions.
